# Adaptation of the First Episode Psychosis Services—Fidelity Scale for Use in New and Small Programs

**DOI:** 10.1111/eip.70151

**Published:** 2026-03-10

**Authors:** Mark Savill, Briana Sepulveda, Lindsay M. Banks, Stephania L. Hayes, Valerie L. Tryon, Christopher Blay, Kathleen E. Burch, Kristin LaCross, Sabrina Ereshefsky, Misha Carlson, Grace Eun Lee, Rachel L. Loewy, Donald E. Addington, Tara A. Niendam

**Affiliations:** ^1^ University of California Davis California USA; ^2^ University of California San Francisco California USA; ^3^ Stanford University Stanford California USA; ^4^ University of Calgary Calgary Alberta Canada

**Keywords:** coordinated specialty care, early intervention services, FEPS‐FS, programme assessment, rural care

## Abstract

**Introduction:**

Fidelity assessments can support healthcare services to deliver care consistent with best practises. However, early psychosis (EP) fidelity assessment tools typically require a volume of service data that is often unavailable to small or new programmes. In this study, we pilot a formative fidelity assessment approach to address these challenges.

**Methods:**

A formative assessment approach to using the First Episode Psychosis Services—Fidelity Scale (FEPS‐FS) was developed to enable the assessment of small and new EP programmes. Over 48 months, EPI‐CAL EP learning health care network programmes completed standard FEPS‐FS fidelity assessments, formative assessments for new programmes, or formative assessments for small programmes, depending upon programme eligibility.

**Results:**

Of 27 remote fidelity assessments completed with EP programmes across California, nine (33.3%) had insufficient service data to complete a standard FEPS‐FS assessment. Utilising the proposed formative assessment approach, one programme met the criteria for a new programme assessment, and seven for a small programme assessment. In the new programme assessment approach, 34 of 36 items could be assessed. In the small programme assessments, a median of 19 items was scored, with a mean FEPS‐FS score range from 3.45 to 4.12.

**Conclusion:**

These findings suggest that a formative approach to fidelity assessment can generate a meaningful amount of data, capture known variability between programmes, and potentially identify areas for service improvement to enhance quality for small and new programmes. However, critical data regarding the delivery of pharmacological and psychosocial care were not captured, highlighting the limitations of the approach.

## 1 Introduction

Early Psychosis (EP) programmes are an effective treatment for individuals with psychosis (Craig et al. 2004; Kane et al. 2016; Bertelsen et al. 2008). Consequently, over the past 10 years, there has been a major expansion of EP programmes worldwide (McGorry 2015; Read and Kohrt 2022). Such programmes typically include pharmacotherapy, psychotherapy, supported employment and education services, case management, peer support, and family services, delivered by a multidisciplinary team (Heinssen et al. 2014). To support programme implementation, fidelity assessments support the delivery of care according to evidence‐based practises (EBP) (Addington et al. 2013). This is important, given that higher fidelity to EBP in healthcare is associated with improved outcomes (Bond and Drake 2020; Rosenblatt et al. 2024).

Different countries and systems have adopted different EP programme fidelity assessment practises (Addington et al. 2022, 2024; Williams et al. 2021), with most incorporating data collected from service user health records. One of the most extensively used EP fidelity tools, the First‐Episode Psychosis Services—Fidelity Scale (FEPS‐FS), requires a chart review of ten individuals enrolled in the programme for at least a year (Addington 2021). However, if the programme has not been providing services for that long or only serves a small number of individuals, there may be insufficient data to meet this requirement. This is not typically a barrier to large, established programmes. However, policy changes supporting the development of evidence‐based programmes for early serious mental illness, such as coordinated specialty care (George et al. 2022; Heinssen et al. 2014), have led to many new programmes being opened in new regions, including programmes set up to provide care for a much smaller census of service users (National Association of State Mental Health Program Directors (NASMHPD) 2018). This is likely to be more prevalent as hub‐and‐spoke and specialist within generalist adaptations to the EP programme model continue to be explored as a method to support care delivery in rural or remote communities (Behan et al. 2017; Pipkin 2021).

Fidelity assessments support the adoption of EBPs (Rapp et al. 2008), and can be critical to discriminating between unplanned drift and intentional adaptations to better meet local needs (Sanetti et al. 2021). When full fidelity assessments are not feasible, formative evaluations may represent a realistic alternative to support new and small programmes' adherence to best‐practise guidelines. Formative evaluations are a rigorous assessment process designed to identify potential and actual influences on the progress and effectiveness of implementation efforts (Elwy et al. 2020; Stetler et al. 2006). Formative evaluations have been used to improve implementation fidelity of interventions across healthcare, including in HIV testing (Bokhour et al. 2015), suicide prevention strategies (Snyder et al. 2025), and mental health youth service hubs (Henderson et al. 2020). Modifying the fidelity assessment process to work with new and small programmes could support more widespread EBP implementation and greater consistency in care delivery. This is particularly important given the challenges of delivering high‐quality EP care in rural communities (Pipkin 2021).

To address these challenges, we developed a formative fidelity assessment approach tailored to new and small programmes. This approach was then trialled across the EPI‐CAL network of California EP programmes to explore its feasibility and utility.

## 2 Materials and Methods

### 2.1 Design

We conducted an observational study of alternative approaches to fidelity assessment for new and small early psychosis programmes.

### 2.2 Setting

All EP programmes in the EPI‐CAL network serving individuals experiencing first episode psychosis (FEP) were eligible for inclusion. EPI‐CAL programmes adopt different eligibility criteria but typically serve individuals aged 14–30 who are experiencing either a psychosis‐spectrum disorder with an onset of psychosis within 2 years or clinical high risk for psychosis (CHRp, Poletti et al. 2024).

### 2.3 Measures

The First‐Episode Psychosis Services—Fidelity Scale (FEPS‐FS) (Addington 2021) is a widely used fidelity measure of team‐based EP care (Durbin et al. 2019; Meneghelli et al. 2023; Tomaskova et al. 2023), reliable for use in remote assessments (Addington et al. 2020), and appropriate for different models of EP care (Addington et al. 2020). The most recent iteration (version 1.1) assesses 36 components (see Table 1), each related to a particular domain of EP care, including team model and function, service access, pharmacotherapy, psychosocial interventions, and assessment practises (Addington et al. 2013). All items are rated on a behaviourally anchored 5‐point scale, with a score of 4 or 5 considered good‐to‐high fidelity to EBP guidelines (Addington et al. 2016).

**TABLE 1:** Items scored across the different FEPS‐FS assessment approaches.

	Item	Item domain	Primary method of rating	Standard assessment	Formative assessment for new programmes	Formative assessment for small programmes	A	B	C
1	Practising Team Leader	Team form/Function	Interviews	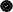	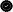	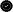			
2	Patient‐to‐Provider Ratio	Team form/Function	Admin Data	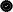	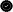	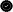			
3	Services Delivered by Team	Team form/Function	Interviews	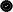	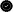	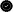			
4	Assigned Case Manager	Team form/Function	Chart Review	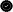	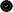		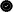		
5	Prescriber Caseload	Team form/Function	Admin Data	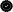	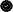	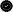			
6	Prescriber Role on Team	Team form/Function	Interviews	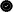	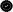	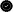			
7	Team Meetings	Team form/Function	Interviews	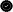	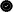	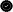			
8	Diagnostic Admission Criteria	Service Access	Interviews	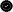	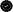	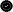			
9	Population Served	Service Access	Admin Data	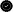	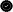	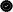			
10	Age Range	Service Access	Admin Data	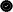	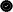	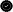			
11	Duration of Programme	Service Access	Interviews	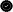	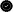	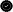			
12	Targeted Outreach	Service Access	Admin Data	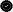	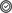	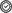			
13	Early Intervention	Service Access	Admin Data	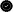	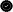		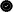		
14	Timely Contact Following Referral	Service Access	Admin Data	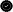	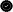		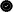		
15	Family Involvement in Assessments	Assessment	Chart Review	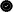	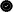		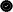		
16	Clinical Assessment	Assessment	Chart Review	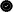	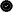		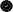		
17	Psychosocial Needs Assessment	Assessment	Chart Review	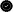	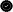		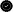		
18	Clinical Care Planning	Assessment	Chart Review	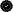	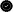		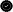		
19	Antipsychotic Medication	Pharmacotherapy	Chart Review	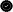	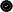				
20	Antipsychotic Dosing	Pharmacotherapy	Chart Review	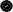	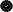				
21	Clozapine Administration	Pharmacotherapy	Admin Data	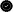	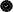		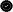		
22	Patient Psychoeducation	Psychosocial Therapy	Chart Review	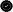	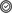				
23	Family Education and Support	Psychosocial Therapy	Chart Review	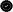	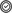				
24	Cognitive Behavioural Therapy (CBT)	Psychosocial Therapy	Chart Review	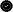	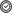				
25	Supporting Health	Multiple Domains	Interviews	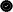	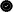	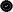			
26	Annual Assessment	Assessment	Chart Review	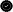					
27	Substance Use Disorder Services	Multiple Domains	Interviews	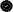	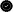	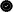			
28	Support Employment (SE)	Multiple Domains	Interviews	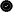	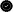	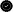			
29	Supported Education (SEd)	Multiple Domains	Interviews	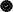	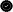	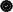			
30	Engagement in Community	Team form/Function	Interviews	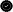	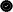	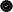			
31	Patient Retention	Team form/Function	Admin Data	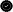	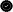				
32	Crisis Intervention Services	Team form/Function	Interviews	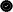	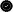	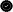			
33	Contact After Inpatient Discharge	Team form/Function	Chart Review					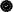	
34	Assuring Fidelity	Team form/Function	Interviews	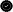	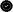	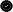			
35	Peer Specialist	Team form/Function	Interviews	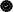	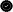	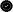			
36	Care Transitions	Team form/Function	Chart Review						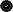

*Note:* 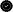 Item Scored Normally.

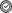
 Item Scored with modified criteria.

A Items to add to ‘Formative Assessments for Small Programmes’ when the programme has 5+ service users currently enrolled.

B Item to add to the assessment when at least five service users have been hospitalised during care.

C Item to add to the assessment when at least 5 service users have been discharged from the programme.

FEPS‐FS fidelity scores are determined based on three data sources, including staff interviews, administrative data, and chart record review data (Addington 2021). The chart record review is completed using three samples, which can include the same service users. These include ‘Chart Review Sample 1’: a randomised sample of 10 current FEP service users enrolled in the programme for at least one year; ‘Chart Review Sample 2’: the last 10 service users discharged from the programme; and ‘Chart Review Sample 3’: the last five service users admitted to a psychiatric hospital after enrolling in care.

**FIGURE 1:**
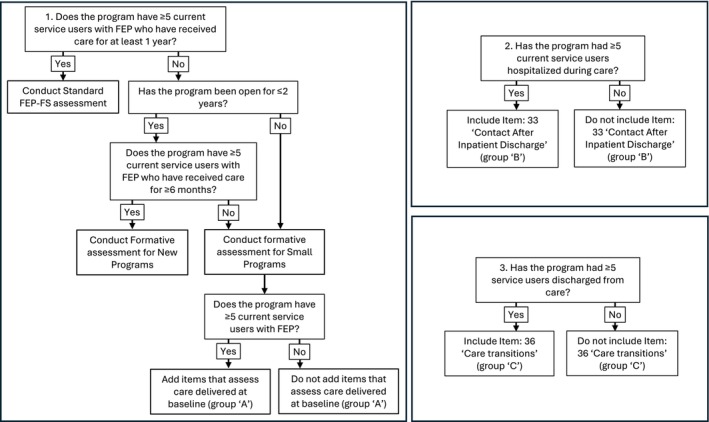
Decision tree used to determine what fidelity assessment approach is appropriate.

To enable the assessment of programmes unable to meet the standard FEP‐FS assessment data requirements, two alternative approaches were developed. These include (1) formative assessments for new programmes and (2) formative assessments for small programmes. A decision tree that helps assessors determine the appropriate assessment is presented in Figure 1, and the items scored in each approach are listed in Table 1.

Across all three fidelity assessment approaches, if the programme had at least five discharged service users, then the item exploring care transitions (item 36) is scored using ‘Chart Review Sample 2’. If the programme has at least five service users admitted to the hospital during care, then the ‘contact after inpatient admission’ item (item 33) is scored using ‘Chart Review Sample 3’.

### 2.4 Standard Fidelity Assessments

In standard fidelity assessments, the administrative and chart record review data are used, consistent with the approach detailed in the FEPS‐FS manual (Addington 2021). In cases where a programme does not have at least 10 current FEP service users enrolled for at least one year, standard FEPS‐FS assessments can still be conducted so long as at least five service users meet these criteria (based on correspondence with the FEPS‐FS author, Dr. Addington). In items assessed on the proportion of individuals that receive a particular service using chart data (i.e., items 4, 14–20, 22–24, 26, and 33; see Table 1), the denominator is the number of individuals included in the review.

### 2.5 Formative Assessments for New Programmes

New programmes are defined as programmes that have a sufficient census of clients in treatment to run a standard fidelity assessment (i.e., more than five) but may not been open long enough to have a sufficient proportion of service users to have received care for at least one year. To meet eligibility for a formative assessment for new programmes, the programme must (1) have been open for less than two years, and (2) have at least five active service users who have received services for over six months.

In formative assessments for new programmes, the eligibility requirements for ‘Chart Review Sample 1 are modified to the following: a randomised sample of up to 10 existing FEP service users enrolled in the programme for at least six months’. To capture this, for FEPS‐FS items scored based on care provided over 12 months (i.e., items 22–24), the number of sessions and the timeline assessed is halved (i.e., five sessions over six months, rather than 10 sessions over 12 months). The item assessing care delivery at the 12‐month point (item 26, annual comprehensive assessment) is not scored. Items scored using ‘Chart Review Sample 1’ that would typically be delivered within the first six months (i.e., items 4, 14–20) are scored following standard FEPS‐FS assessment rules.

Items using administrative data are scored using the standard approach, except for item 12, which evaluates the volume of outreach sessions delivered over the past 12 months. If the programme has been open for less than 12 months, the anchor points are prorated to the length of time the programme has been open.

### 2.6 Formative Assessments for Small Programmes

Programmes that meet the criteria for a formative assessment for small programmes include those ineligible for a standard or new programme assessment. Namely, (1) for programmes that have been open for over two years, they do not have at least five service users in the programme who have received care for at least one year, or (2) for programmes that have been open for less than 2 years, they do not have at least five service users in the programme who have received care for at least six months.

In a small programme assessment, if the programme has at least five active service users enrolled, then the eligibility requirements for ‘Chart Review Sample 1’ are modified to the following: ‘a randomised sample of up to 10 existing FEP service users enrolled in the programme for any duration’. Using these parameters, items that cover the initial stages of care are scored using ‘Chart Review Sample 1’ (items 4, 15–19). Items scored from the chart review concerning care provided over time (items 19, 20, 22–24, 26) are not typically scored. If the programme does not have at least five service users enrolled, no sample is compiled for ‘Chart Review Sample 1’, and these items are not scored.

Regarding the administrative data, items 2, 4, 6, 7, and 8 are all scored normally. If the programme has at least five service users enrolled in care, items 13, 21, and 31 are also scored. Consistent with the formative assessments for new programmes, if the programme has been open for under 12 months, then for item 12, the anchor point is prorated to the duration the programme has been active.

### 2.7 Procedures and Analysis Plan

All EP programmes enrolled in EPI‐CAL receive fidelity assessments every 24 months. Programmes were offered a formative FEPS‐FS assessment if they had an insufficient census to complete a full assessment, after excluding all individuals in the programme that did not meet FEP criteria. New programmes were generally considered to be appropriate for a formative assessment once they had recruited and provided basic training to key programme staff (i.e., the programme lead, clinician, prescriber, case manager if separate from the clinician; and ideally the peer specialist, supported education and employment specialist (SEES), and family specialist if they have the resources to hire them), have the service structure in place to provide care, and be actively taking referrals.

To complete the assessments, fidelity team staff contacted the EP team leaders to explain the process, determine what fidelity assessment approach was appropriate, and train EP programme staff in the fidelity assessment procedures. Each EP programme then took 1–2 months to compile and transfer the administrative and health record data to the fidelity team using a secure portal. Once received, the fidelity team conducted interviews with the programme's team leader, clinician, peer, SEES, and prescriber, as available. These data were then triangulated to determine item scoring. Once the assessment was completed, the fidelity team prepared a comprehensive feedback report for programmes designed to support quality improvement efforts, detailing all summary and item‐level scores, an explanation of how to improve item scores, and a review of programme strengths and opportunities for growth. Finally, the programme reports were reviewed in a feedback session attended by the fidelity assessment team and key programme staff.

To assess the implementation of the proposed formative fidelity assessment approaches, we calculated the total number of FEPS‐FS items that could be scored across each programme, mean programme FEPS‐FS scores, and the proportion of items scored found to meet good‐to‐high fidelity criteria, defined as an item score at least 4 out of 5 (Addington et al. 2016).

## 3 Results

Between 1/11/2021 and 12/6/2024, a FEPS‐FS assessment was completed with 27 unique EPI‐CAL EP programmes. Of these, nine (33.3%) had an insufficient FEP census to complete a standard FEPS‐FS assessment. Programme details and assessment findings of these nine programmes are presented in Table 2. One was an established programme serving a small catchment area; seven were new programmes, of which four were also serving a small catchment area; and one was an established programme in a large catchment area but typically received referrals regarding individuals experiencing CHRp. Individuals experiencing either FEP or CHRp were eligible for care in seven of these programmes, and in two programmes only individuals FEP were eligible. Of those unable to meet full FEPS‐FS assessment criteria, eight met criteria for a small programme assessment, and one for a new programme assessment.

**TABLE 2:** Characteristics of programmes assessed as new or small programmes.

Programme	FEPS‐FS assessment type	Catchment area	Programme structure	Time from programme opening to assessment	FEP/CHRp census during fidelity assessment	Incidence rate in Medi‐Cal population^a^	# of FEPS‐FS items Scored	Mean FEPS‐FS Score	% of Items scored at adherence
Programme 1	Small programme	< 200 000	Standalone	+5 years	3/12	79	20	3.45	55.0%
Programme 2	Small programme	> 1.5 million	Standalone	< 2 years	1/13	571	20	3.75	70.0%
Programme 3	Small programme	< 200 000	Standalone	< 3 years	4/14	19	20	3.45	60.0%
Programme 4	Small programme	< 1 million	Standalone	< 1 year	1/0	172	19	3.84	68.4%
Programme 5	Small programme	< 200 000	Hub and spoke	< 1 year	2/0	29	19	3.94	68.4%
Programme 6	Small programme	< 25 000	Hub and spoke	< 1 year	0/0	4	15	3.80	66.7%
Programme 7	Small programme	< 25 000	Hub and spoke	< 1 year	1/0	10	19	3.95	68.4%
Programme 8^b^	Small programme	> 1.5 million	Standalone	< 2 years	3/N/A	808	19	4.05	73.7%
Programme 9^b^	New programme	> 1.5 million	Standalone	< 2 years	7/N/A	808	34	4.12	76.5%

Abbreviations: CHRp, Clinical High Risk for Psychosis; FEP, First Episode Psychosis; FEPS‐FS First Episode Psychosis Services—Fidelity Scale; N/A, not applicable (i.e., individuals experiencing CHRp do not meet eligibility criteria for these programmes).

^a^ Incidence rate calculated on an expected prevalence of 272 individuals per 100 000 per year (Radigan et al. 2019).

^b^ Programmes were situated in the same county, sharing the same catchment area and incidence rate.

**FIGURE 2:**
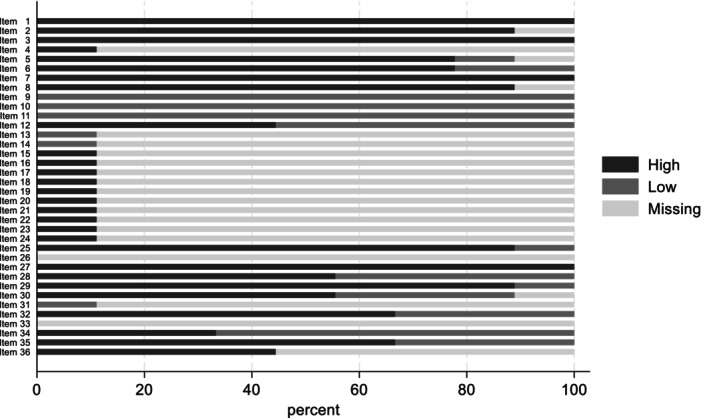
Proportion of items scores, the degree to which each met best practise guidelines.

The proportion of items that could be scored across the nine formative assessments and the degree to which they met good‐to‐high fidelity criteria are presented in Figure 2. In the new programme assessment, 34 of 36 items (94.4%) were assessed. The mean FEPS‐FS score was 4.12, and 76.5% of the items assessed met good to high fidelity criteria. Across small programme assessments, a median of 19 of the 36 (52.8%) FEPS‐FS items were scored (range 15–20). Mean FEPS‐FS programme scores ranged from 3.45 to 4.05. Of the items scored, the proportion of items where programmes achieved good to high fidelity to EBP ranged from 55.0% to 73.7%.

In the small programme assessments, 15 of the 36 items could be scored across all eight programmes (41.7% in total), and 19 items (52.8%) were scored across at least seven programmes. Of those that could be scored across at least seven programmes, 10 items assessed team form or functioning (out of 14 items in this domain), five assessed service access and population‐level interventions (out of seven), and four (out of four) concerned multiple domains, incorporating team form, psychosocial or pharmacological care delivery, and/or assessment. Areas where the small programme evaluations could not address any component of EP care included assessment, pharmacology, and psychosocial interventions.

Across the 15 items that could be consistently scored across both assessment types, only four items were scored at good‐to‐high fidelity (i.e., a 4 or 5) across all programmes assessed (item 1, Team Leader role; Item 3, Services Delivered; Item 7, Function of the Multidisciplinary Team Meeting; and item 27, Substance Use Disorder Services). A further four items were scored at good‐to‐high fidelity in eight of the nine programmes (Item 2, Service User to Provider Ratio; Item 8, Diagnostic Admission Criteria; Item 25, Supporting Health; and Item 29, Supported Education Services). No programmes met good‐to‐high criteria for three items (Item 9, Population Served; Item 10, Age Range; and Item 11, Duration of Programme). In the 14 additional items that could be scored using the new programme approach, the programme scored good‐to‐high fidelity in 11 items.

## 4 Discussion

Across EPI‐CAL programmes that completed a fidelity assessment between 2021 and 2024, 33.3% did not meet the data requirements of a standard FEPS‐FS assessment. This highlights a need for alternative fidelity assessment approaches for programmes with an insufficient census to complete standard assessments. Of the nine programmes that were not eligible for a standard assessment, all were able to complete a formative assessment, supporting the feasibility and generalizability of the approach. It is notable given the wide range of programmes that participated in the study, including those that deliver care as standalone programmes or as part of a hub‐and‐spoke approach, in new and established programmes, and in programmes that serve small and large catchment areas. In the one new programme assessment conducted, most FEPS‐FS items could be scored (34 of 36, 94.4% of items). This was much higher than in the programmes where only a small programme assessment could be completed (range completed: 41.7%–55.6%), highlighting the utility of the new programme approach. However, it is notable that even in the small programme assessments, many items could be scored, suggesting that meaningful findings could be generated even with limited chart data. Across all assessments, opportunities to improve practises were identified, highlighting the utility of the modified assessment from a quality improvement perspective. Finally, the variability of FEPS‐FS scores between programmes (mean score range = 3.45–4.12) indicated that the modified approaches can still capture known variability between programmes. This implies that the approach has criterion validity, which is a key component of evaluating fidelity measure quality (Calsyn 2000). Collectively, these findings highlight the need, feasibility, and utility of adopting a formative approach to fidelity assessments for new and small EP programmes.

Regarding limitations, it is important to note that whilst a broad array of EP programmes was included in the study, all were based in California and participated in EPI‐CAL. Therefore, it will be necessary to test this approach in different systems. Second, it is notable that only one programme met the criteria for a new programme assessment. Therefore, further validation of this approach is necessary. Third, whilst there is substantial overlap between the FEPS‐FS and the version of the tool that assesses CHRp care, known as the Clinical High Risk for Psychosis Services—Fidelity Scale (CHRPS‐FS, Savill et al. 2025), some important differences do exist, which means work to validate formative assessments of CHRp care is necessary. Finally, whilst higher FEPS‐FS scores have been associated with improved treatment outcomes (Rosenblatt et al. 2024), further work is needed to determine if this association still holds in the subset of items used in these formative assessments.

In the assessments, multiple items concerning service access and team form and functioning could be consistently scored. This is particularly important both for new and developing programmes, given the importance of establishing the necessary staff and programme components in place to enable the delivery of EP services (Bello et al. 2017). However, it is important to recognise that the approach was unable to yield information concerning assessment, pharmacotherapy, and psychosocial intervention delivery. These are critical pieces of EP care (Heinssen et al. 2014), and the lack of information in these domains could hamper quality improvement efforts. To mitigate this, in this study, the EPI‐CAL fidelity team elected to provide programmes with provisional scores, where possible. These provisional scores were based on data primarily collected during the interview process and focused on what the programme would very likely achieve based on the implementation of the proposed plan. Whilst these data do not meet the threshold of a standard score, feedback from the EP programme team leaders has been that the information can be invaluable to support quality improvement activities. However, the provisional nature of the ratings needs to be clearly communicated to programmes to mitigate the risk that they are misinterpreted as having achieved full fidelity in a particular domain, leading them not to consider these as possible areas of improvement.

A consequence of these new approaches is that EP programme fidelity assessments may become feasible for smaller rural area programmes. However, this does raise important questions regarding the extent to which adherence to established models developed and refined in urban, often university settings, may be appropriate for programmes in rural areas. Notably, multiple studies that have focused on EP care delivered in rural settings have emphasised the importance of adapting the established model to fit the needs and resources of communities (Cheng et al. 2011; Pipkin 2021; Reznik et al. 2025). In particular, studies have highlighted the greater importance of primary care in rural mental healthcare (Campbell 2005), in addition to the greater degree of connectiveness and cohesion between agencies in rural areas (Welch and Welch 2007). Such features could potentially support more effective EP care access and coordination, which in turn could enable the implementation of adapted service models that are more feasible to implement in rural areas in ways that do not compromise service effectiveness. Going forward, further research into what constitutes best‐practise EP care in rural settings would be highly informative. Fidelity measures are considered an important part of model development (Bond and Drake 2020), and so the modified assessment approach outlined here could be critical to supporting such work.

Given the importance of fidelity assessment to guiding implementation, quality improvement efforts, benchmarking, programme sustainment, and the uptake of new practises (Bond and Drake 2020), it is essential to develop fidelity assessment approaches that can be adopted by a wide range of programmes. This study, therefore, could be an important resource to small and new EP programmes that might otherwise be left behind. Going forward, evaluating the impact of these formative assessments on programme quality improvement efforts will be highly informative. Additionally, exploring how these formative assessments could be expanded to incorporate a more in‐depth qualitative exploration of the barriers and facilitators to implementation, as has been done in some other formative evaluation efforts (i.e., Bokhour et al. 2015), might help newer programmes in particular address barriers to implementation and achieve closer fidelity to best practise over time. Finally, adapting EP fidelity tools such as the FEPS‐FS to enable the full assessment of key EP care components, such as assessment, pharmacotherapy, and psychosocial treatment, will be important to further support small EP programme quality improvement activities and research. In turn, this could help efforts to determine best‐practise EP care models for programmes in rural areas and low‐census programmes.

## Funding

This work was supported by California Department of Health Care Services, Nevada County, One Mind, National Institute of Mental Health (1R01MH120555‐01), Napa County, and Los Angeles County.

## Conflicts of Interest

Ms. Kathleen E. Burch reports having been a paid consultant for ChatOwl Inc., a digital mental health company. Dr. Niendam reports being a cofounder of and shareholder in Safari Health Inc., a digital mental health company. The other authors report no financial relationships with commercial interests.

## Data Availability

The data that support the findings of this study are available from the corresponding author upon reasonable request.
